# Presenteeism in a Dutch hand eczema population—a cross‐sectional survey

**DOI:** 10.1111/cod.12993

**Published:** 2018-04-01

**Authors:** Jart A. F. Oosterhaven, Peter A. Flach, Ute Bültmann, Marie L. A. Schuttelaar

**Affiliations:** ^1^ Department of Dermatology, University of Groningen University Medical Centre Groningen The Netherlands; ^2^ Department of Health Sciences, Community and Occupational Medicine University of Groningen, University Medical Centre Groningen The Netherlands

**Keywords:** absenteeism, hand eczema, occupational, presenteeism

## Abstract

**Background:**

Presenteeism (attending work despite complaints and ill health, which should prompt rest and absence) has been overlooked in the field of hand eczema.

**Objectives:**

To examine the 1‐year prevalence of presenteeism related to hand eczema in a population of hand eczema patients who visited a tertiary referral centre. Secondary objectives: to identify intrinsic/extrinsic reasons for presenteeism and to evaluate associated factors.

**Methods:**

This was a cross‐sectional questionnaire study. Presenteeism was defined as “going to work despite feeling you should have taken sick leave because of hand eczema”. Respondents answered questions about socio‐demographic factors, clinical features, occupational characteristics, and hand eczema related to occupational exposure.

**Results:**

Forty‐one per cent (141/346) of patients who had both worked and had hand eczema during the past 12 months reported presenteeism. The most often reported reasons were: “Because I do not want to give in to my impairment/weakness” (46%) and “Because I enjoy my work” (40%). Presenteeism was associated with: mean hand eczema severity; absenteeism because of hand eczema; improvement of hand eczema when away from work; and high‐risk occupations.

**Conclusions:**

In this study, presenteeism was common and predominantly observed in patients with more severe hand eczema and occupational exposure. The most frequently reported reasons for presenteeism were of an intrinsic nature.

## 1 INTRODUCTION

Hand eczema is one of the most prevalent occupational skin diseases in Europe.^1^, ^2^ In Germany, it is even the most frequently reported occupational disease.^3^ Hand eczema can lead to sickness absenteeism and eventually to job loss and change of profession.^4^, ^5^, ^6^ From other medical conditions, such as allergic rhinitis and arthritis, it is known that sickness absenteeism is often preceded by a phase in which workers try to continue their working activities, while their disease actually hampers their productivity and recovery.^7^, ^8^, ^9^ This phenomenon is called presenteeism: attending work despite complaints and ill health that should prompt rest and absence from work.^10^ Presenteeism can be regarded as a positive concept by workers with chronic conditions who are able to keep working.^11^ However, in both the medical and economic literature, presenteeism is mostly regarded as a negative and counterproductive phenomenon. Presenteeism received little attention for years, but has been increasingly studied in occupational medicine since the start of the 21st century. A recent review highlighted presenteeism as a risk factor for future sickness absence and decreased self‐rated health.^12^ Furthermore, it has been shown that presenteeism may be related to more productivity loss and higher costs than sickness absence in the long term.^13^, ^14^

Despite the fact that hand eczema is frequently caused or aggravated by occupational exposures,^15^ hand eczema‐related presenteeism has hitherto not received much attention. Although a review from 2010 showed a significant impact of occupational contact dermatitis on work activities,^16^ to date only 1 study among patients with hand eczema has addressed presenteeism. Van der Meer et al studied Dutch healthcare professionals with self‐reported hand eczema. They considered presenteeism to be “lost time at work” (in terms of amount and quality of work performed). The 1‐year prevalence of hand eczema in the healthcare professionals was relatively low (12%); of those with hand eczema, 3.1% reported presenteeism and 1.7% reported sickness absence because of hand eczema.^17^ To date, little is known about presenteeism in patients with more severe hand eczema, working in various occupations. Therefore, the aim of this study was to examine the 1‐year prevalence of presenteeism related to hand eczema in a population of hand eczema patients who visited a tertiary referral centre. Secondary objectives were to identify intrinsic or extrinsic reasons for presenteeism, and to evaluate factors associated with the prevalence of presenteeism in hand eczema patients.

## 2 METHODS

### 2.1 Study design

This was a cross‐sectional study carried out at the Department of Dermatology of the University Medical Centre Groningen, a tertiary referral centre for hand eczema. The population of hand eczema patients that visits the department predominantly lives in the 5 northern provinces of The Netherlands (population approximately ⁓3.2 million). Patients were identified by searching electronic patient records from visits between January 1, 2011 and December 31, 2015. Identified patients received a postal questionnaire. In parallel, patients who visited our outpatient clinic and were diagnosed with hand eczema were also recruited. These patients completed the questionnaire digitally on‐site. The questionnaire was developed to assess the prevalence of presenteeism, intrinsic or extrinsic reasons for presenteeism, and factors associated with presenteeism prevalence. Before the start of the study, a pilot study was conducted in 5 hand eczema patients to finalize the questionnaire. The study was reviewed and approved by the Medical Ethical Review Board of the University Medical Centre Groningen (reference METc 2016/169).

### 2.2 Study population and recruitment

Patients of working age, between 20 and 67 years, with hand eczema diagnosed by a dermatologist in the past 5 years were included. The diagnosis was made in accordance with guidelines by Menné et al and Diepgen et al.^18^, ^19^

**Figure 1:**
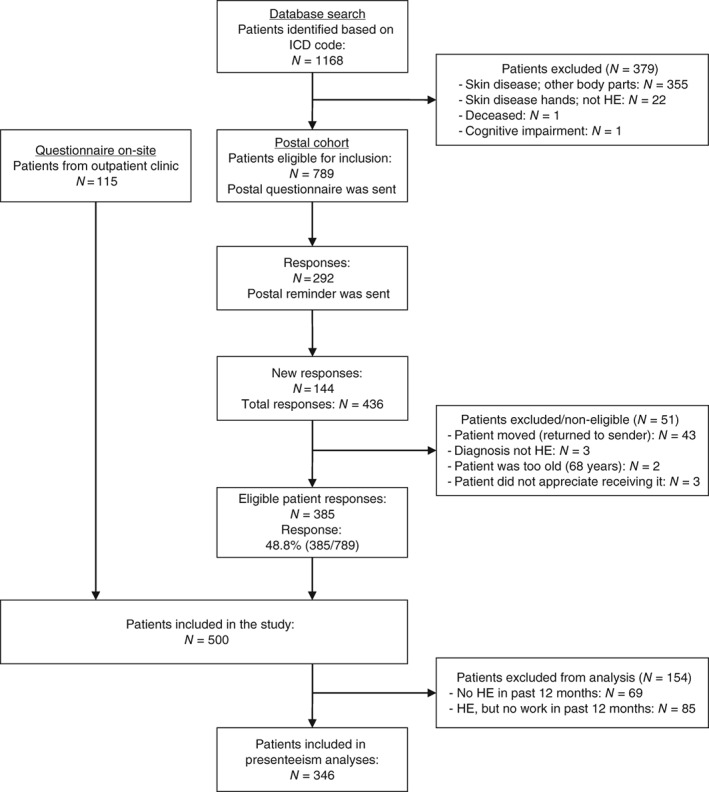
Study flow diagram. HE, hand eczema; ICD, International Classification of Disease

Patients were identified from electronic records by use of the International Classification of Disease (ICD, 10th edition), according to the diagnoses L20 (Atopic dermatitis), L23 (Allergic contact dermatitis), L24 (Irritant contact dermatitis), L25 (Unspecified contact dermatitis), and L30 (Other dermatitis). This yielded a total of 1168 patients. One author (J.O.) manually screened these files and identified a total of 789 patients in whom the diagnosis of hand eczema could be confirmed. The other 379 patients were excluded, mainly because the ICD codes were not specific for hand eczema and the dermatitis occurred on body areas other than the hands in these patients. For a study flow diagram, see Figure 1.

In June 2016, an invitation letter, a questionnaire and a prepaid return envelope were sent to the 789 eligible patients. A reminder was sent after 5 weeks. The recruitment of the on‐site patients took place between June 2016 and March 2017. A total of 115 patients were approached to complete the questionnaire. For data entry by participants on‐site and data entry of the postal questionnaires, the online survey software qualtrics was used (Qualtrics, Provo, Utah; http://www.qualtrics.com). To improve the response rate, 10 gift coupons of €50 were raffled among the participants who responded to the postal questionnaire.

### 2.3 Measurements

All concepts are briefly described below. For a comprehensive overview of the definitions and categorization for the analyses, see Appendix S1. All variables concern the past 12 months unless otherwise indicated.

*Presenteeism*. Patients were asked whether they had both worked and had hand eczema during the past 12 months. In these patients, presenteeism was assessed with the question: “During the past 12 months, did you go to work despite feeling that you should have taken sick leave because of your hand eczema? Yes/no.” The duration of presenteeism was also measured.^20^

*Reasons for presenteeism*. Intrinsic and/or extrinsic reasons for presenteeism were measured with the following question: “You indicated that during the past 12 months you went to work despite feeling that you should have taken sick leave because of your hand eczema. What was the reason for this? (multiple answers possible).” Answer categories were assembled from Johansen et al,^21^ Johns et al,^22^ and Aronsson et al.^10^ Following the pilot study, 2 answer categories were added: “Because I think it is expected of me” and “Because I don't want to give in to my affliction/weakness”.

*Socio‐demographic factors*. Sex; age at questionnaire completion; and education (low/middle or high).

*Clinical features*. First episode of hand eczema ≤18 years;^23^ atopic dermatitis ever;^23^ mean hand eczema severity, which was determined with the photographic guide developed by Coenraads et al;^24^, ^25^ and other longstanding diseases.

*Occupational characteristics*. Type of employment (paid employed/self‐employed);^23^ hours per week; sufficient time at work to perform tasks satisfactorily;^26^ sufficient resources at work to perform tasks satisfactorily;^26^ number of employees; supervising tasks ([non]‐management);^27^ shift work;^27^ high‐risk occupation;^28^, ^29^, ^30^, ^31^, ^32^, ^33^ and monthly income.^21^

*Hand eczema related to occupational exposure*. Absenteeism because of hand eczema; improvement of hand eczema when away from work;^23^ hand eczema related to occupational exposure;^23^ and wet work, which was determined according to the German “Technische Regeln für Gefahrstoffe” (TRGS) 401 criteria^34^ and work by Behroozy et al.^35^

*Covariables*. Frequency of hand eczema ([nearly] all the time or more than once);^23^ months worked; and job loss or early retirement because of hand eczema.

### 2.4 Statistical analysis

Before the analyses were performed, 3 preparatory steps were taken. First, to handle missing values, each completed postal questionnaire was screened. When missing values were found, the sender was contacted by telephone or email to obtain an answer. In this way, all missing data were retrieved. The design of the digital questionnaire did not allow for missing data. Second, respondents and non‐respondents to the postal questionnaire were compared in a non‐response analysis. Third, respondents who completed the postal questionnaire were compared with respondents who completed the questionnaire digitally on‐site. Descriptive statistics were reported by the use of mean and standard deviation for normally distributed variables, and median and interquartile range (IQR) for non‐normally distributed variables. For comparison of normally and non‐normally distributed variables, respectively, Student's *t* test and the Mann‐Whitney *U* test were used. Proportions were compared by use of the *χ* ^2^ test.

Logistic regression analysis was used to evaluate associations with presenteeism prevalence. All variables that were significant in the univariable analysis were entered into a multivariable model. A post hoc sample size calculation for logistic model building was performed. The method based on the work by Peduzzi et al^36^ states that: *N* = 10 *k*/*p*. Here, *p* is the smallest of the proportions of negative or positive cases in the population, and *k* is the number of covariates (the number of independent variables). To build a model with 12 independent variables in a sample with a presenteeism proportion of 0.40, the necessary minimal sample size is *N* = 300. Odds ratios (ORs) with 95% confidence intervals were calculated. Analyses were performed with IBM spss statistics for Windows, Version 23.0 (IBM Corp., Armonk, New York). A *P* value of <.05 was regarded as being statistically significant.

A sensitivity analysis was conducted to examine the influence of income on presenteeism in the multivariable model. Information on income was not provided by all respondents (respondents choosing the options “I don't know” or “I would rather not answer this question”), so, for the analysis, these response options were regarded as missing. As logistic regression analysis only handles complete cases, income was left out of the main multivariable model. In a subgroup analysis, reasons for presenteeism were assessed for the digital questionnaire on‐site group to check whether reasons in this group differed from those in the total group.

## 3 RESULTS

### 3.1 Sample characteristics

Data were collected from 500 participants, aged between 20 and 67 years. The response to the postal questionnaire was 48.8%. The non‐response analysis showed no sex difference, but non‐respondents were significantly younger than respondents: 40 years (IQR 29.0‐52.0) vs 49 years (IQR 36.0‐58.0); *P* < .01. Respondents to the digital questionnaire on‐site were significantly younger than respondents to the postal questionnaire. Also, during the past 12 months, their hand eczema prevalence was higher, they had more severe hand eczema, they worked during fewer months, and they called in sick more often because of their hand eczema (Table 1).

**Table 1:** Basic characteristics of the total study population (*N* = 500) and a comparison between respondents to the postal questionnaire and respondents to the digital questionnaire on‐site

	Total, *N* = 500	Respondents to postal questionnaire, *n* = 385	Respondents to digital questionnaire on‐site, *n* = 115	*P* value
Socio‐demographics				
Female sex, % (*n*/*n*_total_)	55.8 (279/500)	57.7 (222/385)	49.6 (57/115)	.13
Age (years), median (IQR)	46.5 (34.0‐57.0)	49.0 (36.0–58.0)	40.0 (27.0‐53.0)	**<.001**
Education, % (*n*/*n*_total_)				.14
Low/middle	75.4 (377/500)	73.8 (284/385)	80.9 (93/115)	
High	24.6 (123/500)	26.2 (101/385)	19.1 (22/115)	
Clinical features, % (*n*/*n*_total_)				
First episode of HE ≤18 years	74.8 (374/500)	76.4 (294/385)	69.6 (80/115)	.14
Atopic dermatitis, ever	47.6 (238/500)	45.5 (175/385)	54.8 (63/115)	.09
HE during the past 12 months	86.2 (431/500)	82.3 (317/385)	99.1 (114/115)^a^	**<.001**
HE (nearly) all the time or more than once	86.3 (372/431)	84.5 (268/317)	91.2 (104/114)	.08
Mean HE severity				**<.001**
No HE during past 12 months	11.2 (56/500)	14.5 (56/385)^a^	0 (0/115)^a^	
Almost clear	25.4 (127/500)	29.9 (115/385)	10.4 (12/115)	
Moderate	38.2 (191/500)	35.6 (137/385)	47.0 (54/115)	
Severe	20.6 (103/500)	17.1 (66/385)	32.2 (37/115)	
Very severe	4.6 (23/500)	2.9 (11/385)	10.4 (12/115)	
Occupational characteristics				
Not employed during past 12 months, % (*n*/*n*_total_)	19.7 (85/431)	19.9 (63/317)	19.3 (22/114)	.81
Paid employed, % (*n*/*n*_total_)	63.6 (274/431)	63.8 (199/317)	65.8 (75/114)	.81
Months worked, median (IQR)	12 (12‐12)	12 (12–12)	12 (11‐12)	**.03**
Work hours per week, mean (±SD)	31.0 (±12.5)	30.8 (±12.3)	31.5 (±12.9)	.67
Self‐employed, % (*n*/*n*_total_)	16.7 (72/431)	17.4 (55/317)	14.9 (17/114)	.81
Months worked, median (IQR)	12 (10‐12)	12 (10‐12)	12 (10.5‐12)	.33
Work hours per week, mean (±SD)	37.5 (±20.3)	37.4 (±19.2)	37.6 (±24.2)	.98
High‐risk occupation, % (*n*/*n*_total_)^b^	43.6 (151/346)	42.9 (109/254)	45.7 (42/92)	.71
Monthly income, % (*n*/*n*_total_)				.89
Low	45.7 (118/258)	46.2 (86/186)	44.4 (32/72)	
Middle/high	54.3 (140/258)	53.8 (100/186)	55.6 (40/72)	
HE related to occupational exposure, % (*n*/*n*_total_)				
Worked during the past 12 months while having had HE during the past 12 months	80.3 (346/431)	80.1 (254/317)	80.7 (92/114)	.89
Absenteeism because of HE during the past 12 months	16.2 (56/346)	12.2 (31/254)	27.2 (25/92)	**.001**
Improvement of HE when away from work	45.4 (157/346)	48.0 (122/254)	38.0 (35/92)	.11
HE related to occupational exposure	50.3 (174/346)	52.4 (133/254)	44.6 (41/92)	.22
Wet work	46.2 (160/346)	45.7 (116/254)	47.8 (44/92)	.81
Job loss or early retirement because of HE during the past 12 months	4.0 (20/500)	3.1 (12/385)	7.0 (8/115)	.10
Presenteeism, % (*n*/*n*_total_)				
Prevalence during the past 12 months	40.8 (141/346)	38.2 (97/254)	47.8 (44/92)	.11
Duration of >42 days	17.0 (24/141)	17.5 (17/97)	15.9 (7/44)	.81

HE, hand eczema; IQR, interquartile range; SD, standard deviation.

a Thirteen respondents responded inconsistently to the questions on the presence of hand eczema in the past 12 months and mean severity over the past 12 months. These patients were not included in further analyses.

b See Appendix S4 for an overview of the occupations in the sample.

### 3.2 Presenteeism prevalence and reasons for presenteeism

In the analyses of presenteeism prevalence, 346 respondents were included because they had both worked and had hand eczema during the past 12 months. A total of 141 (40.8%) respondents reported that they had experienced presenteeism during the past year. No significant difference in presenteeism prevalence was observed between the respondents to the digital and postal questionnaires.

Table 2 shows the reported reasons for presenteeism with a differentiation between 5 intrinsic and 11 extrinsic reasons. In total, 505 answers were provided by the 141 respondents who reported presenteeism (175 intrinsic; 308 extrinsic; 22 other reasons that were not categorized). The median number of reasons reported was 3 (IQR 2‐5). Two intrinsic reasons for presenteeism were mentioned most often: “I do not want to give in to my impairment/weakness” (46.1% of respondents) and “I enjoy my work” (39.7% of respondents). Notably, 22.7% of respondents went to work because they were “afraid of losing their job”. Self‐employed respondents (*n* = 29) were more inclined to choose extrinsic reasons, with financial motives and irreplaceable responsibilities being the most often reported reasons (total reasons 85; 28 intrinsic reasons [33%], and 57 extrinsic reasons [67%]). Paid employed respondents (*n* = 112) chose intrinsic reasons more often than self‐employed individuals (total reasons 398; 147 intrinsic reasons [37%], and 251 extrinsic reasons [63%]). See Appendix S2 for all reported reasons for both groups.

**Table 2:** Intrinsic and extrinsic reasons for presenteeism in 141 workers with hand eczema

Reasons for presenteeism	Intrinsic/extrinsic motivation	*n* (%)
Because …		
… I do not want to give in to my impairment/weakness	Intrinsic	65 (46.1)
… I enjoy my work	Intrinsic	56 (39.7)
… I think it is expected of me	Extrinsic	52 (36.9)
… I do not want to burden my colleagues	Extrinsic	41 (29.1)
… I do not want to be considered lazy or unproductive	Extrinsic	37 (26.2)
… financially I cannot afford taking sick leave	Extrinsic	32 (22.7)
… I am afraid of losing my job	Extrinsic	32 (22.7)
… my pride keeps me from calling in sick	Intrinsic	28 (19.9)
… my employer expects it of me	Extrinsic	25 (17.7)
… no one else can take over my responsibilities	Extrinsic	25 (17.7)
… I have appointments with clients/patients	Extrinsic	23 (16.3)
… I need to catch up on a lot of work if I have been sick	Extrinsic	17 (12.1)
… going to work is good for my health	Intrinsic	14 (9.9)
… I do not want to be suspected of cheating	Extrinsic	13 (9.2)
… I want to maintain my social network	Intrinsic	12 (8.5)
… I feel ashamed to call in sick	Extrinsic	11 (7.8)
Other reasons^a^		22 (15.6)

The total percentage exceeds 100% because subjects were permitted to choose multiple reasons.

a Other reported reasons were: “I don't ‘feel sick’” (6); “I am self‐employed” (2); “other work could (temporarily) replace my normal work” (2); “it never occurred to me to call in sick for hand eczema” (2); “I work with livestock” (2); “I didn't want to get in trouble over calling in sick; I could easily work from home; I can't sit still; I don't have enough insurance and can't afford employees; re‐organization at work; I don't consider hand eczema a reason for calling in sick; people don't take eczema seriously so I don't want to be considered a fraud; it doesn't match my character to call in sick”.

### 3.3 Factors associated with presenteeism

In a univariable logistic regression analysis (Table 3), significant associations were found between presenteeism and variables from all 4 domains (socio‐demographics, clinical features, occupational characteristics, and hand eczema related to occupational exposure). Significant variables were included in the multivariable model. For income, see sensitivity analyses.

**Table 3:** Univariable logistic regression analysis for the association of factors within 4 domains with presenteeism

	Total % (*n*/*n*_total_)	Presenteeism % (*n*/*n*_total_)	Crude OR (95%CI)	*P* value
Socio‐demographics				
Sex				.82
Male	48.3 (167/346)	40.1 (67/167)	1.00 (ref.)	
Female	51.7 (179/356)	41.3 (74/179)	1.05 (0.69‐1.62)	
Age group (y)				.17
20‐35	33.2 (115/346)	46.1 (53/115)	1.00 (ref.)	
36‐50	32.4 (112/346)	33.9 (38/112)	0.60 (0.35‐1.03)	
51‐67	34.4 (119/346)	42.0 (50/119)	0.85 (0.51‐1.42)	
Education				**.01**
Low/middle	74.6 (258/346)	44.6 (115/258)	1.00 (ref.)	
High	25.4 (88/346)	29.5 (26/88)	**0.52 (0.31‐0.87)**	
Clinical features				
First episode of HE				.71
≤18 years	29.5 (102/346)	39.2 (40/102)	1.00 (ref.)	
>18 years	70.5 (244/346)	41.4 (101/244)	1.10 (0.68‐1.76)	
Atopic dermatitis ever				**.02**
No	50.0 (173/346)	34.7 (60/173)	1.00 (ref.)	
Yes	50.0 (173/346)	46.8 (81/173)	**1.66 (1.08‐2.56)**	
Mean HE severity				**<.001**
Almost clear	30.3 (105/346)	13.3 (14/105)	1.00 (ref.)	
Moderate	43.1 (149/346)	42.3 (63/149)	**4.76 (2.49‐9.12)**	
Severe	22.5 (78/346)	70.5 (55/78)	**15.54 (7.39‐32.71)**	
Very severe	4.0 (14/346)	64.3 (9/14)	**11.70 (3.42‐40.01)**	
Other longstanding diseases				.57
No	68.5 (237/346)	41.8 (99/237)	1.00 (ref.)	
Yes	31.5 (109/346)	38.5 (42/109)	0.87 (0.55‐1.39)	
Occupational characteristics				
Type of employment				.93
Paid employed	79.2 (274/346)	40.9 (112/274)	1.00 (ref.)	
Self‐employed	20.8 (72/346)	40.3 (29/72)	0.98 (0.58‐1.66)	
Mean weekly working hours				.39
≤23	24.6 (85/346)	47.1 (40/85)	1.00 (ref.)	
24‐35	31.2 (108/346)	38.0 (41/108)	0.69 (0.39‐1.23)	
≥36	44.2 (153/346)	39.2 (60/153)	0.73 (0.43‐1.24)	
Sufficient time at work				**.004**
No	9.8 (34/346)	64.7 (22/34)	1.00 (ref.)	
Yes	90.2 (312/346)	38.1 (119/312)	**0.34 (0.16‐0.71)**	
Sufficient resources at work				**.009**
No	4.6 (16/346)	75.0 (12/16)	1.00 (ref.)	
Yes	95.4 (330/346)	39.1 (129/330)	**0.21 (0.07‐0.68)**	
Number of employees				.61
Self‐employed	11.8 (41/346)	39.0 (16/41)	1.00 (ref.)	
1‐9	15.3 (53/346)	49.1 (26/53)	1.51 (0.66‐3.44)	
10‐99	29.5 (102/346)	40.2 (41/102)	1.05 (0.50‐2.21)	
≥99	43.4 (150/346)	38.7 (58/150)	0.99 (0.49‐2.00)	
Supervising tasks				.67
Non‐management	81.2 (281/346)	40.2 (113/281)	1.00 (ref.)	
Middle management/executive	18.8 (65/346)	43.1 (28/65)	1.13 (0.66‐1.94)	
Shift work				.66
No	79.2 (274/346)	40.1 (110/274)	1.00 (ref.)	
Yes	20.8 (72/346)	43.1 (31/72)	1.13 (0.67‐1.91)	
High‐risk occupation				**.002**
No	56.4 (195/346)	33.3 (65/195)	1.00 (ref.)	
Yes	43.6 (151/346)	50.3 (76/151)	**2.02 (1.31‐3.14)**	
Income				**.04**
Low	45.7 (118/258)	50.0 (59/118)	1.00 (ref.)	
Middle/high	54.3 (140/258)	37.1 (52/140)	**0.59 (0.36‐0.97)**	
HE related to occupational exposure				
Absenteeism because of HE				**<.001**
No	83.8 (290/346)	32.1 (93/290)	1.00 (ref.)	
Yes	16.2 (56/346)	85.7 (48/56)	**12.7 (5.78‐28.0)**	
Improvement of HE when away from work				**<.001**
No	54.6 (189/346)	31.7 (60/189)	1.00 (ref.)	
Yes	45.4 (157/346)	51.6 (81/157)	**2.29 (1.48‐3.55)**	
HE related to occupational exposure				**.002**
No	49.7 (172/346)	32.6 (56/172)	1.00 (ref.)	
Yes	50.3 (174/346)	48.9 (85/174)	**1.98 (1.28‐3.06)**	
Wet work				.05
No	53.8 (186/346)	36.0 (67/186)	1.00 (ref.)	
Yes	46.3 (160/346)	46.3 (74/160)	1.53 (0.99‐2.35)	

CI, confidence interval; HE, hand eczema; OR, odds ratio.

In the multivariable regression model (Table 4), more severe hand eczema; absence because of hand eczema in the past 12 months, hand eczema that improved when away from work; and working in a high‐risk occupation were significantly associated with presenteeism prevalence.

**Table 4:** Multivariable logistic regression model for presenteeism

	Mutually adjusted OR (95%CI)	*P* value
Mean HE severity		**<.001**
Almost clear	1.00 (ref.)	
Moderate	**5.52 (2.63‐11.6)**	
Severe	**17.6 (7.32‐42.4)**	
Very severe	**6.80 (1.57‐29.4)**	
Absenteeism because of HE		**<.001**
No	1.00 (ref.)	
Yes	**9.96 (4.06‐24.5)**	
Improvement of HE when away from work		**.009**
No	1.00 (ref.)	
Yes	**2.20 (1.22‐3.96)**	
High‐risk occupation		**.009**
No	1.00 (ref.)	
Yes	**2.14 (1.21‐3.78)**	
Sufficient time at work		.15
No	1.00 (ref.)	
Yes	0.44 (0.15‐1.33)	
Sufficient resources at work		.19
No	1.00 (ref.)	
Yes	0.34 (0.07‐1.71)	
HE related to exposure at work		.24
No	1.00 (ref.)	
Yes	1.42 (0.79‐2.53)	
Atopic dermatitis ever		.53
No	1.00 (ref.)	
Yes	1.19 (0.69‐2.06)	
Education		.60
Low/middle	1.00 (ref.)	
High	1.20 (0.61‐2.32)	

CI, confidence interval; HE, hand eczema; OR, odds ratio.

### 3.4 Sensitivity and subgroup analyses

A higher income was significantly associated with a lower presenteeism prevalence in the univariable analysis. Eighty‐eight respondents chose not to disclose information about their income or could not answer this question. When income was added to the multivariable model in Table 4, it was no longer significantly associated with presenteeism. The effect of having a high‐risk occupation attenuated and became non‐significant (*P* = .07, *n* = 258). The other significant independent variables remained significant; see Appendix S3.

In a subgroup analysis, the distribution of reasons for presenteeism in the digital questionnaire on‐site group was assessed. This showed a very similar ranking to that in the whole group. The same 2 intrinsic reasons for presenteeism were most frequently reported: “Because I do not want to give in to my impairment/weakness” (57.7%) and “Because I enjoy my work” (40.9%). See Appendix S3.

## 4 DISCUSSION

In this study, we found a 1‐year prevalence of 41% for presenteeism because of hand eczema. Intrinsic reasons for presenteeism were common, and the phenomenon was strongly associated with severity and occupational characteristics.

More than 40% of the study population indicated that they had experienced presenteeism because of hand eczema in the past 12 months. This proportion is strikingly higher than the low percentage (3%) that was found by van der Meer et al in their study in Dutch healthcare workers.^17^ The discrepancy could partly be explained by the probably mild hand eczema severity of their homogeneous working population, which might have minimized the interference with work, as compared with our occupationally heterogeneous patient population with much more severe hand eczema. Another explanation could be the different definition of presenteeism that was used. We focused on whether respondents felt that they should have called in sick although they did not (subjectively), instead of asking for lost time at work in terms of amount and quality of work performed (a somewhat more objective measure). However, the difference is large, indicating that the phenomenon is indeed quite common in our patient population. A comparison with presenteeism in patients with other chronic diseases is difficult, mainly because many studies measure presenteeism as a decline in productivity or quality of work, using various measurement instruments. The problem here is that there are still no generally accepted measurement instruments with which to assess presenteeism. A large range of existing instruments were found to be insufficiently validated.^37^ With these instruments, a wide range of presenteeism (19%‐79%) was found in studies in several chronic diseases, including systemic lupus erythematosus, spondyloarthritis, inflammatory bowel disease, low back pain, and rheumatic arthritis.^38^, ^39^, ^40^, ^41^, ^42^, ^43^, ^44^ A recent study with a definition more comparable to that used in the present study was performed by d'Errico et al in a sample of the European working population. The authors asked >30 000 workers the question “Over the past 12 months did you work when you were sick?”. For 6 health problems (insomnia, stomach pain, headache, upper arm pain, back pain, and wounds), they found rather similar percentages of presenteeism, ranging between 42.3% (back pain) and 52.2% (insomnia).^45^ The percentage found in our population is slightly lower. This could be explained by the fact that we did not mark every respondent who worked with hand eczema as having presenteeism. We incorporated an additional aspect in our definition of presenteeism by including the phrase “… despite feeling you should have taken sick leave because of your hand eczema”. This could be regarded by respondents as a proxy for expected productivity loss (“I am going to work although I think I will be less productive because of my hand eczema today”). It could also be regarded as resulting from fear that their hands would become much worse if they worked, regardless of whether this was at the expense of their productivity (“I am going to work although I think my hand eczema will get worse due to my working activities today”). These 2 explanations might influence each other greatly and may very often coexist. New and better‐validated instruments are needed to assess productivity loss caused by presenteeism.^37^, ^46^ Once these reliable and valid instruments become available, future studies should focus on determining whether presenteeism in patients with hand eczema actually causes productivity loss.

A notable finding in our study is the intrinsic nature of the most often reported reasons for presenteeism. This was also found by Robertson et al^47^ and partly by Johansen et al (“enjoying work” was the second most reported reason in their study, after “don't want to burden my colleagues”).^21^ It seems that presenteeism in our patients is often self‐imposed. This is an important finding, especially in patients with occupational hand eczema. It is easily assumed that the tendency to attend work, although being detrimental to hand eczema, is mainly financially driven or forced by third parties. Our study, however, shows that, although feelings of external pressure along with financial motives exist, intrinsic reasons are even more important for many individuals. This does not apply to self‐employed individuals, in whom extrinsic reasons are more common than in paid employees. Patients should be made aware of the fact that presenteeism can lead to deterioration of hand eczema. Along with their occupational physician, patients need to determine their occupational exposure and the feasibility of using adequate protection against hazardous exposure in their work. To enable adequate assessment of this, the dermatologist should provide the occupational physician with information about the sensitization profile from patch testing and about the tolerance of the skin of the hands to irritants, and its repair capacity after exposure to irritants.^35^

Although intrinsic reasons are most often reported, it is important to note that more than 1 in 4 respondents reported “Because I am afraid of losing my job” as a reason for presenteeism. This figure is much higher than the 4% reported by Johansen et al in a sample of the general working population with unspecified medical conditions in Norway and Sweden.^21^ It seems that having hand eczema causes patients much worry about being able to keep their jobs.

Strong associations were found between presenteeism prevalence and mean hand eczema severity, absenteeism because of hand eczema, improvement of hand eczema when away from work, and working in a high‐risk occupation. The association of these factors provides a picture of a predominance of patients who experience hand eczema‐related presenteeism because of more severe hand eczema that is at least partly caused or aggravated by work. Many of these patients had already called in sick on several occasions because of hand eczema, but probably only to give their hands a little rest. From other diseases, we know that this kind of behaviour can eventually cause long‐term absence if it is continued for some time.^12^ Interestingly, in the group of patients with very severe hand eczema, the adjusted OR for presenteeism prevalence is lower than in the severe group. This is possibly because the patients with such very severe disease eventually do call in sick for a longer time (data not shown). Notably, self‐employment was not associated with presenteeism. This is probably related to the importance of intrinsic motivations that drive workers to attend work while having hand eczema, regardless of employment status.

A limitation of our study is that we chose not to incorporate psychosocial factors, mainly because this would further increase the amount of items in the questionnaire. By asking our question about presenteeism specifically in relationship to hand eczema, we mainly addressed the health state aspect of presenteeism. However, the question can be raised of whether to look at presenteeism as a health state phenomenon (originating mainly from the medical condition) or rather as behaviour (implying that a choice is made, supported by psychosocial characteristics). A recent meta‐analysis found evidence for both.^48^ This is in agreement with Brooks et al, who stated that presenteeism should be approached as a complex system, with incorporation of variables from both the health state and a behavioural point of view.^49^

Another possible limitation can be found in the definition of high‐risk occupations. For certain occupations, working activities are quite well known and similar between different workers in this branch (eg, hairdressers and bakers). For other occupations, much more variation exists (eg, in healthcare workers). To precisely determine whether an individual is working in a high‐risk occupation, the job content, working process and exposure levels are more important than the job title.

Methodologically, a possible limitation could be common method variance; we measured the outcome and all associated variables with the same self‐report questionnaire. Furthermore, it is possible that non‐response bias in the postal questionnaire group could have influenced the results of this study. Unfortunately, we only had sex and age of the postal non‐respondents available, so it is not possible to draw conclusions about the presence of non‐response bias.

Data were collected with 2 different methods (postal and digital). The postal and digital on‐site respondents were combined for analysis. Age, mean severity of hand eczema, absenteeism because of hand eczema and months worked during the past 12 months significantly differed between the postal and digital on‐site respondents. This was expected regarding severity and absenteeism, as the digital respondents were all patients who sought care from a dermatologist when completing the questionnaire, whereas the postal respondents had visited the department at some point during the past 5 years. Nevertheless, presenteeism prevalence was not significantly different between the groups. This, along with similarities in several other variables (eg, sex, education, occupational characteristics, and, especially, whether respondents had both worked and had hand eczema during the past 12 months), led us to combine the respondent groups. Also, reasons for presenteeism in the digital respondent group showed a very similar ranking to those of the whole group in the subgroup analysis.

Finally, we did not control for a possible influence of certain lifestyle factors, such as smoking, high body mass index, or alcohol use, which have been identified as possible risk factors for presenteeism.^50^

In this study, we have shown that presenteeism is a common phenomenon in patients with more severe hand eczema. The most frequently reported reasons for presenteeism were of an intrinsic nature. Dermatologists and occupational physicians should pay attention to presenteeism to provide more individually targeted care for hand eczema patients.

## CONFLICT OF INTEREST

There was no funding and the authors report no conflicts of interest, either actual or perceived.

## Supporting information


**Appendix S1**. Variable definitions and grouping.

**Appendix S2**. Reasons based on employment status.

**Appendix S3**. Sensitivity and subgroup analyses.

**Appendix S4**. Frequencies of occupation.
