# Supplementary material for: Presenteeism in a Dutch hand eczema population—a cross‐sectional survey
Source: Contact Dermatitis. 2018 Apr 1;79(1):10–9. doi: 10.1111/cod.12993 (PMC6001425; doi:10.1111/cod.12993)
Supplement: Supplementary file 1 — Appendix S1. Variable definitions and grouping. [file COD-79-10-s002.docx]

**Supplement S1**

**Variables: definitions and categorization**

**To establish hand eczema and work and employment status**

Question D1 derived from the Nordic Occupational Skin Questionnaire (NOSQ), modified:

*Have you had hand eczema during the past 12 months?*

- No
- Yes

A self-formulated question:

*Did you work during the past 12 months?*

- No I did not work
- Yes, I worked as employee
- Yes, I was self-employed

**Definition and operationalization of the dependent variable**

Presenteeism was assessed with a modified version of the presenteeism question by Johansen et al (1) to focus on hand eczema:

*During the past 12 months, did you go to work despite feeling that you should have taken sick leave because of your hand eczema?*

- Yes
- No

Duration of presenteeism was assessed with the question:

*How many days in total during the past 12 months did you go to work despite thinking that you should have reported in sick considering your hand eczema?*(2)

- Less than 7 days
- Between 7 and 21 days
- Between 22 and 42 days
- More than 42 days

These values for duration were chosen based on the current rules of the Dutch employee insurance agency on sickness absence.

**Definition of independent variables and categorization**

| **Independent variable** | **Question and response options** | **Categorization** | **Reference** |
| --- | --- | --- | --- |
| *Socio-demographic factors* | | | |
| Sex | Are you…?   - Male (1) - Female (2) |  | None |
| Age | What is your age? (Please round off to whole years) | 1: 20-35  2: 36-50  3: 51-67 | None |
| Education | What is your highest level of completed education?   - No degree (1) - Primary education, elementary school, special education (1) - Preparatory secondary vocational education (1) - Lower secondary general education (1) - Senior secondary vocational education (2) - Higher general secondary education (2) - Higher professional education (3) - Academic higher education (3) | 1: Low/middle  2: High | None |
| *Clinical features* | | | |
| First episode of hand eczema ≤ 18 years | When did you first get eczema on your hands?   - Below 6 years of age (1) - Between 6 and 14 years of age (1) - Between 15 and 18 years of age (1) - Above 18 years of age (2) | 1: ≤ 18 years  2: > 18 years | NOSQ question D6, modified (3) |
| Atopic dermatitis, ever | Have you ever had an itchy rash that has been coming and going for at least 6 months, and at some time has affected skin creases? (by skin creases we mean folds of elbows, behind the knees, fronts of ankles, under buttocks, around the neck, ears, or eyes)   - No (0) - Yes (1) - Don’t know (0) | 0: No  1: Yes | NOSQ question A1(3) |
| Mean hand eczema severity | On average, which group did your hand eczema match over the past 12 months?   - I did not have hand eczema (1) - Almost clear (2) - Moderate (3) - Severe (4) - Very severe (5) |  | Photographic guide by Coenraads et al. combined with a question from Hald et al (4, 5) |
| Other longstanding diseases | Do you have a longstanding disease or handicap (other than hand eczema)?   - No (0) - Yes (1) |  | None |
| *Occupational characteristics* | | | |
|  |  |  |  |
| Work hours per week | How many hours per week did you work? (Please round off to whole hours) | 1: ≤ 23  2: 24-35  3: ≥ 36 | None |
| Sufficient time at work | Did you have sufficient time to perform your working tasks satisfactorily during the past 12 months?   - Always (1) - Usually (1) - Usually not (0) - Never (0) | 0: No  1: Yes | Modified from Aronsson and Hansen (6, 7) |
| Sufficient resources at work | Did you have sufficient resources to perform your working tasks satisfactorily during the past 12 months?   - Always (1) - Usually (1) - Usually not (0) - Never (0) | 0: No  1: Yes | Modified from Aronsson and Hansen (6, 7) |
| Number of employees | How many workers are employed in the company where you worked during the past 12 months?   - I am self-employed and work alone - 1-9 employees - 10-99 employees - 99+ employees |  | None |
| Supervising tasks | Did you have a supervising position during the past 12 months?   - No, I am self-employed and work alone (0) - Yes, I was only supervising (1) - Yes, but I also performed the same work as employees (1) - No, I was not supervising (0) | 0: Non-management  1: Middle management/ executive | NEA 2014, question 4c, modified (8) |
| Shift work | Did you work in shifts during the past 12 months?   - Yes, often (1) - Yes, sometimes (1) - No (0) | 0: No  1: Yes | NEA 2014, question 2n, modified (8) |
| High-risk occupation | What is your occupation? Open-ended question.  High-risk occupation was defined as evident exposure to allergens and/or evident exposure to wet work/friction with a high probability of developing hand eczema:  Agricultural workers / gardeners  Bakers/pastry makers  Beauty specialists/nail stylists  Butchers/slaughterhouse workers  Canning and fish processing industry workers  Construction workers/carpenters  Cooks/kitchen workers/vegetable processers  Dental technicians  Fitters  Florists  Hairdressers  Healthcare workers  Housekeepers/cleaners  Metal surface processers  Painters and varnishers  Plasterers  Tanners  Tile setters and terazzo workers  All other occupations were regarded as non-high-risk. | 0: non-high-risk  1: high-risk | To determine high-risk occupations we used six publications.(9-14) We combined the results from these publications and two experts (MLA Schuttelaar and PJ Coenraads) reached consensus on the definitive list of high-risk occupations.  See also Supplement S4. |
| Monthly income | What was your monthly net income during the past 12 months (during the months you actually worked)? (We mean take-home pay, after reduction of taxes)   - I don’t know (99) - I would rather not answer this question (99) - Less than €750 (1) - €750 - €1000 (1) - €1000 - €1500 (1) - €1500 - €2000 (2) - €2000 - €2500 (2) - €2500 - €3000 (2) - €3000 - €3500 (2) - More than €3500 (2) | 1: Low  2: Mid/high  99: Missing | Question by Johansen et al, modified (1) |
| *Hand eczema related to occupational exposure* | | | |
| Hand eczema related to occupational exposure | Have you noticed that contact with certain materials, chemicals or anything else in your work makes your eczema worse?   - No (0) - Yes (1) - Don’t know (0) | 0: No  1: Yes | NOSQ question F1, modified (3) |
| Wet work | To define wet work, we asked three questions:   - *On an average working day, while working, how many hours do your hands come into direct contact with water, fluids and/or moist products? Never (0)/less than 0.5 hours (1)/0.5 – 1 hour (2)/1 -2 hours (3)/more than 2 hours (4).* - *On an average working day, while working, how many hours do you wear gloves that are impermeable to fluids? Never (0)/less than 0.5 hours (1)/0.5 – 1 hour (2)/1 -2 hours (3)/more than 2 hours (4).* - *On an average working day, while working, how often do you wash your hands? Never (0)/less than 5 times (1)/5 – 10 times (2)/10 – 20 times (3)/more than 20 times (4).* | Wet work was defined as minimally 2 or more from answer category 3, or 1 from answer category 4 | Questions based on the German Technische Regeln für Gefahrstoffe (TRGS) 401 criteria and an article by Behroozy et al. (15, 16) |
| Absenteeism because of hand eczema | How many days during the past 12 months did you call in sick because of your hand eczema?   - I did not call in sick because of hand eczema (0) - Less than 7 days (1) - Between 7 and 42 days (1) - More than 42 days (1) | 0: No  1: Yes | None |
| Improvement of hand eczema when away from work | Does your hand eczema improve when you are away from your normal work (for example weekends or longer periods)?   - No (0) - Yes, sometimes (1) - Yes, usually (1) - Don’t know (0) | 0: No  1: Yes | NOSQ question F4, modified (3) |

**Definition of covariables**

| **Covariable** | **Question and response options** | **Categorization** | **Reference** |
| --- | --- | --- | --- |
| *Clinical features* | | | |
| Hand eczema (nearly) all the time or more than once | How often have you had eczema on your hands during the past 12 months?   - Only once and for less than two weeks (0) - Only once but for two weeks or more (0) - More than once (1) - (Nearly) all the time (1) | 0: No  1: Yes | NOSQ question D4, modified (3) |
| *Occupational characteristics* | | | |
| Months worked | How many months did you work during the past 12 months? (Please round off to whole months) |  | None |
| *Hand eczema related to occupational exposure* | | | |
| Job loss or early retirement because of hand eczema during the past 12 months | During the past 12 months, did you become unemployed or were you forced to go into early retirement because of your hand eczema?   - No (0) - Yes (1) |  | None |

NEA, Nationale Enquête Arbeidsomstandigheden voor werknemers (Dutch Questionnaire on Working Conditions for Employees); NOSQ, Nordic Occupational Skin Questionnaire.

**References**

1. Johansen V, Aronsson G, Marklund S. Positive and negative reasons for sickness presenteeism in Norway and Sweden: a cross-sectional survey. BMJ Open 2014;4:e004123-2013-004123.

2. Taloyan M, Aronsson G, Leineweber C, et al. Sickness presenteeism predicts suboptimal self-rated health and sickness absence: a nationally representative study of the Swedish working population. PLoS One 2012;7:e44721.

3. Susitaival P, Flyvholm MA, Meding B, et al. Nordic Occupational Skin Questionnaire (NOSQ-2002): a new tool for surveying occupational skin diseases and exposure. Contact Dermatitis 2003;49:70-76.

4. Coenraads PJ, Van Der Walle H, Thestrup-Pedersen K, et al. Construction and validation of a photographic guide for assessing severity of chronic hand dermatitis. Br J Dermatol 2005;152:296-301.

5. Hald M, Veien NK, Laurberg G, Johansen JD. Severity of hand eczema assessed by patients and dermatologist using a photographic guide. Br J Dermatol 2007;156:77-80.

6. Aronsson G, Gustafsson K. Sickness presenteeism: prevalence, attendance-pressure factors, and an outline of a model for research. J Occup Environ Med 2005;47:958-966.

7. Hansen CD, Andersen JH. Going ill to work--what personal circumstances, attitudes and work-related factors are associated with sickness presenteeism? Soc Sci Med 2008;67:956-964.

8. Centraal Bureau voor de Statistiek (CBS), Nederlandse Organisatie voor Toegepast Natuurwetenschappelijk Onderzoek (TNO). NEA: Nationale Enquête Arbeidsomstandigheden voor werknemers, 2014. Available at: <http://www.monitorarbeid.tno.nl/dynamics/modules/SFIL0100/view.php?fil_Id=125> (last accessed 13 June 2017).

9. Halkier-Sørensen L. Notified occupational skin diseases in Denmark. Important exposure sources, occupations and trades. The course from notification to compensation and socio-economical aspects. Contact Dermatitis 1996;35 (suppl 1):1-120.

10. Diepgen TL. Occupational skin-disease data in Europe. Int Arch Occup Environ Health 2003;76:331-338.

11. Skoet R, Olsen J, Mathiesen B, et al. A survey of occupational hand eczema in Denmark. Contact Dermatitis 2004;51:159-166.

12. Diepgen TL. Occupational skin diseases. J Dtsch Dermatol Ges 2012;10:297-313; quiz 314-5.

13. Schwensen JF, Friis UF, Menné T, Johansen JD. One thousand cases of severe occupational contact dermatitis. Contact Dermatitis 2013;68:259-268.

14. Diepgen TL, Andersen KE, Chosidow O, et al. Guidelines for diagnosis, prevention and treatment of hand eczema. J Dtsch Dermatol Ges 2015;13:e1-22.

15. Bundesanstalt für Arbeitsschutz und Arbeitsmedizin. TRGS 401: Risks resulting from skin contact - identification, assessment, measures, 2008. Available at: <https://www.baua.de/DE/Angebote/Rechtstexte-und-Technische-Regeln/Regelwerk/TRGS/TRGS-401.html> (last accessed April 12 2017).

16. Behroozy A, Keegel TG. Wet-work Exposure: A Main Risk Factor for Occupational Hand Dermatitis. Saf Health Work 2014;5:175-180.
