# Supplementary material for: Presenteeism in a Dutch hand eczema population—a cross‐sectional survey
Source: Contact Dermatitis. 2018 Apr 1;79(1):10–9. doi: 10.1111/cod.12993 (PMC6001425; doi:10.1111/cod.12993)
Supplement: Supplementary file 2 — Appendix S2. Reasons based on employment status. [file COD-79-10-s004.docx]

**Supplement S2**

**Reasons for presenteeism based on employment status**

Intrinsic and extrinsic reasons for presenteeism in N=112 paid employed workers with hand eczema (sorted descending, ‘other’ reasons excluded).

| **Reasons for presenteeism** | **In- / extrinsic motivation** | **N (%)** |
| --- | --- | --- |
| Because… | | |
| … I do not want to give in to my impairment/weakness | Intrinsic | 55 (49.1) |
| … I enjoy my work | Intrinsic | 46 (41.1) |
| … I think it is expected of me | Extrinsic | 46 (41.1) |
| … I do not want to burden my colleagues | Extrinsic | 37 (33.0) |
| … I do not want to be considered lazy or unproductive | Extrinsic | 35 (31.3) |
| … I am afraid of losing my job | Extrinsic | 27 (24.1) |
| … my employer expects it of me | Extrinsic | 24 (21.4) |
| … my pride keeps me from calling in sick | Intrinsic | 23 (20.5) |
| … financially I cannot afford taking sick leave | Extrinsic | 21 (18.8) |
| … no one else can take over my responsibilities | Extrinsic | 14 (12.5) |
| … I have appointments with clients/patients | Extrinsic | 13 (11.6) |
| … going to work is good for my health | Intrinsic | 13 (11.6) |
| … I do not want to be suspected of cheating | Extrinsic | 12 (10.7) |
| … I need to catch up on a lot of work if I have been sick | Extrinsic | 11 (9.8) |
| … I feel ashamed to call in sick | Extrinsic | 11 (9.8) |
| … I want to maintain my social network | Intrinsic | 10 (8.9) |

Total percentage exceeds 100% because subjects were permitted to choose multiple reasons.

Intrinsic and extrinsic reasons for presenteeism in N=29 self-employed workers with hand eczema (sorted descending, ‘other’ reasons excluded).

| **Reasons for presenteeism** | **In- / extrinsic motivation** | **N (%)** |
| --- | --- | --- |
| Because… | | |
| … financially I cannot afford taking sick leave | Extrinsic | 11 (37.9) |
| … no one else can take over my responsibilities | Extrinsic | 11 (37.9) |
| … I have appointments with clients/patients | Extrinsic | 10 (34.5) |
| … I do not want to give in to my impairment/weakness | Intrinsic | 10 (34.5) |
| … I enjoy my work | Intrinsic | 10 (34.5) |
| … I need to catch up on a lot of work if I have been sick | Extrinsic | 6 (20.7) |
| … I think it is expected of me | Extrinsic | 6 (20.7) |
| … I am afraid of losing my job | Extrinsic | 5 (17.2) |
| … my pride keeps me from calling in sick | Intrinsic | 5 (17.2) |
| … I do not want to burden my colleagues | Extrinsic | 4 (13.8) |
| … I do not want to be considered lazy or unproductive | Extrinsic | 2 (6.9) |
| … I want to maintain my social network | Intrinsic | 2 (6.9) |
| … my employer expects it of me | Extrinsic | 1 (3.4) |
| … going to work is good for my health | Intrinsic | 1 (3.4) |
| … I do not want to be suspected of cheating | Extrinsic | 1 (3.4) |
| … I feel ashamed to call in sick | Extrinsic | 0 (0.0) |

Total percentage exceeds 100% because subjects were permitted to choose multiple reasons.

Intrinsic and extrinsic reasons for presenteeism in N=141 paid employed and self-employed workers with hand eczema. Head-to-head comparison.

| **Reasons for presenteeism** | **In- / extrinsic motivation** | **Paid employed N_total_ = 112**  **N (%)** | **Self- employed N_total_ = 29**  **N (%)** | **p-value** |
| --- | --- | --- | --- | --- |
| Because… | | | | |
| … I do not want to give in to my impairment/weakness | Intrinsic | 55 (49.1) | 10 (34.5) | 0.21 |
| … I enjoy my work | Intrinsic | 46 (41.1) | 10 (34.5) | 0.53 |
| … I think it is expected of me | Extrinsic | 46 (41.1) | 6 (20.7) | 0.05 |
| … I do not want to burden my colleagues | Extrinsic | 37 (33.0) | 4 (13.8) | 0.07 |
| … I do not want to be considered lazy or unproductive | Extrinsic | 35 (31.3) | 2 (6.9) | **<0.01** |
| … I am afraid of losing my job | Extrinsic | 27 (24.1) | 5 (17.2) | 0.62 |
| … my employer expects it of me | Extrinsic | 24 (21.4) | 1 (3.4) | **0.03** |
| … my pride keeps me from calling in sick | Intrinsic | 23 (20.5) | 5 (17.2) | 0.80 |
| … financially I cannot afford taking sick leave | Extrinsic | 21 (18.8) | 11 (37.9) | **0.04** |
| … no one else can take over my responsibilities | Extrinsic | 14 (12.5) | 11 (37.9) | **<0.01** |
| … I have appointments with clients/patients | Extrinsic | 13 (11.6) | 10 (34.5) | **<0.01** |
| … going to work is good for my health | Intrinsic | 13 (11.6) | 1 (3.4) | 0.30 |
| … I do not want to be suspected of cheating | Extrinsic | 12 (10.7) | 1 (3.4) | 0.30 |
| … I need to catch up on a lot of work if I have been sick | Extrinsic | 11 (9.8) | 6 (20.7) | 0.11 |
| … I feel ashamed to call in sick | Extrinsic | 11 (9.8) | 0 (0.0) | 0.08 |
| … I want to maintain my social network | Intrinsic | 10 (8.9) | 2 (6.9) | 0.99 |

Total percentage exceeds 100% because subjects were permitted to choose multiple reasons.
