# Supplementary material for: Presenteeism in a Dutch hand eczema population—a cross‐sectional survey
Source: Contact Dermatitis. 2018 Apr 1;79(1):10–9. doi: 10.1111/cod.12993 (PMC6001425; doi:10.1111/cod.12993)
Supplement: Supplementary file 3 — Appendix S3. Sensitivity and subgroup analyses. [file COD-79-10-s003.docx]

**Supplement S3**

**Sensitivity and subgroup analyses**

*Multivariable model – whole study group*

The sensitivity analysis concerns the model from Table 4 of the original article, including income (Table 1). Confidence intervals are broader because of the smaller sample size owing to the missing values for income.

Table 1. Multivariable logistic regression model for presenteeism including income. Whole group.

|  | **Mutually adjusted OR**  **(95% CI)** | ***p*-Value** |
| --- | --- | --- |
| Mean HE severity |  | **<0.001** |
| Almost clear | 1.00 (ref.) |  |
| Moderate | **3.68 (1.65–8.21)** |  |
| Severe | **13.1 (4.83–35.4)** |  |
| Very severe | **10.9 (1.42–83.0)** |  |
| Absenteeism because of HE |  | **<0.001** |
| No | 1.00 (ref.) |  |
| Yes | **9.40 (3.44–25.7)** |  |
| Improvement of HE when away from work |  | **0.009** |
| No | 1.00 (ref.) |  |
| Yes | **2.50 (1.26–4.98)** |  |
| High-risk occupation |  | 0.07 |
| No | 1.00 (ref.) |  |
| Yes | 1.82 (0.96–3.46) |  |
| Sufficient resources at work |  | 0.08 |
| No | 1.00 (ref.) |  |
| Yes | 0.17 (0.02–1.26) |  |
| HE related to occupational exposure |  | 0.36 |
| No | 1.00 (ref.) |  |
| Yes | 1.38 (0.70–2.74) |  |
| Sufficient time at work |  | 0.41 |
| No | 1.00 (ref.) |  |
| Yes | 0.57 (0.16–2.12) |  |
| Income |  | 0.66 |
| Low | 1.00 (ref.) |  |
| Mid/high | 0.86 (0.45–1.66) |  |
| Atopic dermatitis ever |  | 0.81 |
| No | 1.00 (ref.) |  |
| Yes | 1.08 (0.58–2.02) |  |
| Education |  | 0.85 |
| Low/middle | 1.00 (ref.) |  |
| High | 1.08 (0.51–2.30) |  |

CI, confidence interval; HE, hand eczema; OR, odds ratio.

*Digital questionnaire on-site group – reasons for presenteeism*

The subgroup analysis concerns the group that completed the questionnaire digitally on-site. In Table 1 from the original article we show that the postal and digital on-site groups are quite similar, except for age, mean severity, absenteeism because of hand eczema, and months worked during the past 12 months. In this subgroup analysis, we checked the distribution of reasons for presenteeism in the digital questionnaire group to assess whether they responded similar to this question, when compared to the whole group.
In total, 161 answers were provided by 44 respondents with presenteeism. The median number of reasons reported was 3 (IQR 2-5). Table 2 shows a very similar pattern compared to the whole group (Table 2 in original article). The same two intrinsic reasons for presenteeism were most frequently reported.

Table 2. Intrinsic and extrinsic reasons for presenteeism in N=44 workers with hand eczema. Digital questionnaire on-site group.

| **Reasons for presenteeism** | **In- / extrinsic motivation** | **N (%)** |
| --- | --- | --- |
| Because… | | |
| … I do not want to give in to my impairment/weakness | Intrinsic | 25 (57.5) |
| … I enjoy my work | Intrinsic | 18 (40.9) |
| … I do not want to burden my colleagues | Extrinsic | 16 (36.4) |
| … I think it is expected of me | Extrinsic | 15 (34.1) |
| … I do not want to be considered lazy or unproductive | Extrinsic | 14 (31.8) |
| … I am afraid of losing my job | Extrinsic | 10 (22.7) |
| … financially I cannot afford taking sick leave | Extrinsic | 9 (20.5) |
| … I have appointments with clients/patients | Extrinsic | 9 (20.5) |
| … my pride keeps me from calling in sick | Intrinsic | 7 (15.9) |
| … my employer expects it of me | Extrinsic | 7 (15.9) |
| … no one else can take over my responsibilities | Extrinsic | 7 (15.9) |
| … I do not want to be suspected of cheating | Extrinsic | 7 (15.9) |
| … I feel ashamed to call in sick | Extrinsic | 5 (11.4) |
| … I need to catch up on a lot of work if I have been sick | Extrinsic | 3 (6.8) |
| … going to work is good for my health | Intrinsic | 3 (6.8) |
| … I want to maintain my social network | Intrinsic | 3 (6.8) |
| Other reasons* |  | 5 (11.4) |

Total percentage exceeds 100% because subjects were permitted to choose multiple reasons.

* Other reasons: *I am self-employed (2); I don’t “feel sick” (2); other work could (temporarily) replace my normal work.*
