# Supplementary material for: Presenteeism in a Dutch hand eczema population—a cross‐sectional survey
Source: Contact Dermatitis. 2018 Apr 1;79(1):10–9. doi: 10.1111/cod.12993 (PMC6001425; doi:10.1111/cod.12993)
Supplement: Supplementary file 4 — Appendix S4. Frequencies of occupation. [file COD-79-10-s001.docx]

**Supplement S4**

**Occupations in the sample (high/low risk, sorted descending)**

| **High risk** | **N (%) (N_total_=346)** |
| --- | --- |
| Healthcare workers | 68 (19.7) |
| Housekeepers/cleaners | 21 (6.1) |
| Cooks/kitchen workers/vegetable processers | 10 (2.9) |
| Hairdressers | 10 (2.9) |
| Construction workers/carpenters | 8 (2.3) |
| Metal surface processers | 8 (2.3) |
| Painters and varnishers | 7 (2.0) |
| Agricultural workers / gardeners | 6 (1.7) |
| Tile setters and terazzo workers | 4 (1.2) |
| Beauty specialists/nail stylist | 3 (0.9) |
| Florists | 2 (0.6) |
| Bakers/pastry makers | 1 (0.3) |
| Dental technicians | 1 (0.3) |
| Plasterers | 1 (0.3) |
| Tanners | 1 (0.3) |
| Butchers/slaughterhouse workers | 0 |
| Canning and fish processing industry workers | 0 |
| Fitters | 0 |
|  |  |
| **Non high-risk** |  |
| Office worker | 103 (29.8) |
| Shop assisstant (clerk)/cashier | 15 (4.3) |
| Mechanic | 15 (4.3) |
| Catering employee | 14 (4.0) |
| Driver | 10 (2.9) |
| Apothecary worker | 3 (0.9) |
| Factory worker | 7 (2.0) |
| Machine operator | 3 (0.9) |
| Security worker | 2 (0.6) |
| Swimming pool worker | 2 (0.6) |
| Laundry worker | 2 (0.6) |
| Seaman | 2 (0.6) |
| Mail deliverers | 2 (0.6) |
| Sculptor | 1 (0.3) |
| Acupuncturist | 1 (0.3) |
| Soldier | 1 (0.3) |
| Firefighter | 1 (0.3) |
| Pest control worker | 1 (0.3) |
| Beekeeper | 1 (0.3) |
| Laboratory worker | 1 (0.3) |
| Fittness instructor | 1 (0.3) |
| Unclassifiable | 7 (2.0) |
